# Editorial: Digital health applications of social robots

**DOI:** 10.3389/frobt.2025.1773450

**Published:** 2026-01-08

**Authors:** Cedomir Stanojevic, Casey Bennett, Jennifer Piatt, Selma Šabanović

**Affiliations:** 1 Department of Health and Wellness Design, School of Public Health – Bloomington, Indiana University, Bloomington, IN, United States; 2 Department of Health Informatics, College of Computing and Digital Media, DePaul University, Chicago, IL, United States; 3 Indiana University-Indianapolis, School of Health and Human Sciences, Department of Graduate Health Professions, Indianapolis, IN, United States; 4 School of Informatics and Computing, Indiana University, Bloomington, IN, United States

**Keywords:** AI, digital health (DH), DH applications, digital therapeutics (DTX), healthcare, patient care, patient engagement

## 1 Introduction

The intersection of Artificial Intelligence (AI), robotics and healthcare is gaining prominence, particularly the use of socially assistive robots (SARs) (Stanojevic et al., 2023; Bennett et al., 2022a; Bennett et al., 2024; Bennett et al., 2018; Collins et al., 2024; Stanojevic et al., 2025; Bennett et al., 2017; Hsu and Šabanović, 2013; Perumandla et al., 2025; Šabanović et al., 2013; Toombs et al., 2024). As one of the emerging forms of digital therapeutics (DTx) tools, SARs can improve patient care and engagement alongside technology (i.e., mobile phones, wearables, smart home devices) (Stanojevic et al., 2023). This transformative shift in healthcare introduces new tools, while also shaping how we think about care delivery, patient engagement and the everyday ecologies in which health and wellbeing are supported.

SARs, as a DTx tool, can benefit patients directly through interactive capabilities as well as part of the broader “Digital Health” (DH) systems. Data from sensors onboard the robot, for example, can help identify in-home behaviors, activity patterns and health status of patients remotely (Stanojevic et al., 2023). Furthermore, linking the robotic sensor data to other DH system components (e.g., mobile phones, wearables, smart home devices, virtual/augmented reality) enables the SAR to function as part of an Internet of Things (IoT) ecosystem, creating a broader long-term picture of patient health outcomes in daily life beyond the clinic (Bennett et al., 2022b). In this way, SARs are not simply “devices” placed in a home or clinical setting; they become active, sensing, adaptively-responding components of a larger networked care infrastructure. Such a paradigm shift is transformative for robots in healthcare.

In 2021, $57.2 billion was invested in DH systems around the world (Rock Health, 2022), recognizing the promise this concept holds for aiding in delivery and care management. DH systems traditionally include a blend of various technologies, AI and physiological biomarkers, which combined have the potential to provide support for individuals with a broad array of health conditions. DTx is a more specific set of technology-enabled interventions within the broader DH sphere intended to produce a measurable therapeutic effect (Goldsack et al., 2019), empowering both patients and healthcare providers. This dual therapeutic and informational role positions DTx, and SARs within it, as a crucial bridge between moment-to-moment patient experience and longer-term clinical decision-making.

The main challenge with social robots within DH systems is that the sheer volume and limited oversight of different robotic platforms is hindering validation efforts from technical, clinical, system and privacy standpoints, thus consequently slowing widespread adoption of SARs as treatment tools. Regulatory frameworks, clinical trial designs and implementation strategies are struggling to keep pace with rapid technological development. Therefore, the goal of this Research Topic was to solicit contributions that demonstrate the use of social robots in DH applications, either via empirical research studies or via proposed theoretical solutions, as well as to position SARs more clearly as part of rigorously validated, ethically grounded, and clinically meaningful DTx ecosystems. The Research Topic features several papers that move us closer to that goal.

## 2 Contents of the special issue

Authors were encouraged to submit papers for this Research Topic that contribute to advancing the integration of social robots with other DH components, so as to enhance the overall understanding of patient health outcomes. This Research Topic aimed to gather insights focused on overcoming the obstacles related to validating and adopting technologies for DTx in general (robots or otherwise), particularly concerning technical, clinical, system and privacy aspects. We were especially interested in work that does not treat SARs as stand-alone tools, but rather situates them within multi-device ecosystems, complex care networks, clinical organizational structures, and everyday life contexts.

The authors who submitted work for this special topic have aptly responded to the call. It is evident from the work provided that SARs are increasingly being considered as DTx tools with the potential to be integrated into the broader DH systems. The papers published in this Research Topic cover usage of SARs across the lifespan, from children in acute care settings to older adults living at home or in long-term care. Papers framed as perspectives, systematic literature reviews and original research give both breadth and depth to the subject matter that is being explored. One of the key, commendable themes that connects all these works is the participatory nature of research being done in this field. The construction, refinement and deployment of robots is conducted through approaches that actively involve end users, caregivers, clinicians and other stakeholders in the design of robot systems for healthcare. Furthermore, in the SARs field, particularly, there is a strong and growing recognition of the need for theory-driven, context-aware and trust-sensitive design if these systems are to move from promising prototypes to reliable DTx components of digital health. In other words, “safety” is of paramount importance if social robots or other AI systems are going to be used for healthcare purposes in the real world. That is a common thread throughout all the papers included in this Research Topic, whether it stems from careful alignment of robot behavior during design *a priori* or incorporating appropriate guardrails for the AI after deployment *a posteriori*.

### 2.1 Highlights of the collected papers in this issue

Boudouraki et al. use the NASSS (Non-adoption, Abandonment, Scale-up, Spread, Sustainability) implementation framework to show how robotics and sensor platforms in care homes are shaped by complex organizational, regulatory and material constraints, as well as competing stakeholder values. Building on their previous work and engaging healthcare stakeholders, the authors introduce the concept of an adaptive system where sensor technologies are integrated into robotic platforms through a series of interactive demonstrations. This research provides a better understanding of SARs as a DTx tool that would support and fit within broader DH systems, particularly in settings such as care homes where organizational realities and resource constraints are ever-present.

Klier and Lugrin proposed a wellbeing-centered conceptual model for SAR applications with cognitively healthy older adults, grounded in Self-Determination Theory (SDT) and Positive Emotions, Engagement, Relationships, Meaning, and Accomplishment (PERMA) model, to systematically align robot behaviors with basic psychological needs and flourishing rather than only deficit reduction. The contribution of this work to the field of social robotics is very significant, as the use of SARs in treatment should be aligned with strength-based, person-centered approaches that are moving away from traditional deficit-based frameworks. If SARs are to become effective DTx tools and part of validated DH systems, the use and design of these robots should be informed by models such as SDT addressing autonomy, competence and relatedness of patients and PERMA, with special focus on positive emotions, engagement, relationships, meaning and accomplishment. Presenting a step forward in the right direction bridging clinical practice and robotic design this article explicitly connects psychological theory, wellbeing outcomes and SAR behavior design.

Extending these insights into an acute care environment, Foster et al. describe the participatory designed, AI-enhanced planning and real-world usability testing of a SAR deployed in pediatric emergency departments. The concrete design guidelines for clinical SAR applications focus on managing children’s pain and distress during intravenous procedures. Achieving successful autonomous adaptiveness of the robotic agent is commendable in itself, and even more commendable when we consider what this DTx tool is used for. Beyond technical challenges, which were addressed through iterative refinement, this team successfully integrated the SAR into clinical workflows, with positive responses from children, caregivers and healthcare providers. Their work exemplifies how SARs can be woven into busy clinical environments in ways that respect existing routines while opening new possibilities for emotionally attuned, AI-supported care.

Complementing these perspectives, Olatunji et al. conducted a synthesis of literature on design considerations for SARs that support older adults’ healthy activities at home, emphasizing user-centered design, integration within existing care networks and a roadmap for embedding SARs into DH systems. Systematic reviews often offer much-needed context and build arguments and directions for certain points of view. A somewhat obvious conclusion, that SARs need to be designed for specific needs and be accepted by and useful to the users, is a much-needed reiteration in this field that paves the way to the third conclusion these authors make - integration of SARs into the healthcare ecosystem and care network. Digital health systems need this type of thinking if adoption and validation of these systems is to be done. The emphasis on design according to specific needs of the DTx tool, with adaptive abilities, is another aspect that would provide more usefulness of the tool itself and increase the likelihood of sustained, meaningful engagement.

Along these lines, Gul et al. systematically review how trust in SARs is conceptualized and measured with older adults, highlighting methodological gaps and introducing the Subjective Objective Trust Assessment Human Robot Interaction (SOTA-HRI) framework combining subjective and objective trust indicators as a prerequisite for sustained real-world adoption. A critical aspect that hinders ubiquitous SAR use as DTx is that most previous studies were conducted in lab settings with subjective self-report measures like questionnaires, interviews and surveys to measure the trust of older adults in SARs. Also, convenience sampling was identified as a concern, where many studies reviewed focus on healthy older adults without age-related disabilities. Furthermore, findings reported by these authors align with previous claims that the lack of standardization is one of the key problems in adoption of DH systems and is emblematic of deeper issues in the way studies are conducted (Mathews et al., 2019). Gul et al. analyzed factors influencing trust and found no consensus on any single factor that exerted greater influence. This paper should be observed as a call to action in the field of SARs and, perhaps, a pointer toward the next Research Topic topic focused specifically on trust, ethics and real-world validation.

Collectively, these papers motivate and inform work to showcase SARs as DTx tools, and tackle the intertwined challenges of ecosystem integration, real-time data use, AI-driven adaptation, and the validation, acceptability and trustworthiness required for SARs to become reliable components of next-generation DH systems. They frame SARs not as isolated gadgets, but as socio-technical interventions whose therapeutic impact depends on careful attention to context, wellbeing outcomes, ecosystem integration, robust autonomy and trustworthy interaction. As editors of this Research Topic, we see these contributions as both a reflection of how far the field has come and an invitation to continue pushing forward, toward SARs that are clinically rigorous, ethically grounded, person-centered and deeply embedded within the digital health systems that increasingly shape contemporary care.
